# Empagliflozin and Dapagliflozin Increase Na^+^ and Inward Rectifier K^+^ Current Densities in Human Cardiomyocytes Derived from Induced Pluripotent Stem Cells (hiPSC-CMs)

**DOI:** 10.3390/cells11233707

**Published:** 2022-11-22

**Authors:** María Dago, Teresa Crespo-García, Anabel Cámara-Checa, Josu Rapún, Marcos Rubio-Alarcón, María Marín, Juan Tamargo, Ricardo Caballero, Eva Delpón

**Affiliations:** 1 Department of Pharmacology and Toxicology, School of Medicine, Instituto de Investigación Sanitaria Gregorio Marañón, Universidad Complutense de Madrid, 28040 Madrid, Spain; 2 Centro de Investigación Biomédica en Red Enfermedades Cardiovasculares (CIBERCV), Instituto de Salud Carlos III, 28029 Madrid, Spain

**Keywords:** empagliflozin, dapagliflozin, sodium current, inward rectifier current, human cardiomyocytes

## Abstract

Dapagliflozin (dapa) and empagliflozin (empa) are sodium-glucose cotransporter-2 inhibitors (SGLT2is) that reduce morbidity and mortality in heart failure (HF) patients. Sodium and inward rectifier K^+^ currents (I_Na_ and I_K1_), carried by Nav1.5 and Kir2.1 channels, respectively, are responsible for cardiac excitability, conduction velocity, and refractoriness. In HF patients, Nav1.5 and Kir2.1 expression are reduced, enhancing risk of arrhythmia. Incubation with dapa or empa (24-h,1 µM) significantly increased I_Na_ and I_K1_ densities recorded in human-induced pluripotent stem cell-cardiomyocytes (hiPSC-CMs) using patch-clamp techniques. Dapa and empa, respectively, shifted to more hyperpolarized potentials the I_Na_ activation and inactivation curves. Identical effects were observed in Chinese hamster ovary (CHO) cells that were incubated with dapa or empa and transiently expressed human Nav1.5 channels. Conversely, empa but not dapa significantly increased human Kir2.1 currents in CHO cells. Dapa and empa effects on I_Na_ and I_K1_ were also apparent in Ca-calmodulin kinase II-silenced CHO cells. Cariporide, a Na^+^/H^+^ exchanger type 1 (NHE1) inhibitor, did not increase I_Na_ or I_K1_ in hiPSC-CMs. Dapa and empa at therapeutic concentrations increased I_Na_ and I_K1_ in healthy human cardiomyocytes. These SGLT2is could represent a new class of drugs with a novel and long-pursued antiarrhythmic mechanism of action.

## 1. Introduction

Dapagliflozin (dapa) and empagliflozin (empa) are sodium-glucose cotransporter-2 inhibitors (SGLT2is) used in treatment of type 2 diabetes (T2DM) [1]. Both improve cardiovascular outcomes, as they significantly decrease hospitalizations and mortality in patients with heart failure (HF) with reduced ejection fraction, regardless of presence or absence of T2DM [1,2,3]. Furthermore, it has more recently been demonstrated that empa and dapa also significantly reduce cardiovascular death and hospitalization for HF with preserved ejection fraction in patients with or without T2DM [4,5]. Previous data and meta-analysis have suggested that dapa in particular may exert antiarrhythmic effects that decrease incidence of ventricular arrhythmia and fatal or resuscitated sudden cardiac death [6,7].

At the cellular level, empa (1–10 µM) inhibits the late component of the Na^+^ current (I_NaL_) recorded in ventricular myocytes from patients with aortic stenosis [8] as well as in transfected cells and cardiomyocytes dissociated from mouse models of HF with preserved and reduced ejection fraction [9,10]. Conversely, acute application of empa does not modify peak Na^+^ current (I_Na_) at any stimulation frequency [9,10]. In a rat model of metabolic syndrome, in which I_Na_ and K^+^ currents were increased and decreased, respectively, administration of dapa ameliorated electrical remodeling produced by the metabolic syndrome [11]. 

In human cardiomyocytes, I_Na_, generated by Nav1.5 channels, is responsible for cardiac action potential depolarization, while the inward rectifier current (I_K1_), generated by Kir2.1, channels is responsible for setting resting membrane potential and modulating the late phase of repolarization and action potential duration [12]. Therefore, I_Na_ and I_K1_ critically determine excitability, impulse propagation, and refractoriness. Peak I_Na_ and inward rectifier K^+^ current decrease in cardiomyocytes from patients with HF and reduced ejection fraction by several mechanisms that are probably concomitant [13,14,15,16,17]. This electrical remodeling contributes to potentiation of arrhythmia by reducing excitability and intraventricular conduction velocity [18]. However, it is unknown whether SGLT2is modulate the human peak I_Na_ and I_K1_. Here, we tested the effects of 24 h incubation with empa or dapa at therapeutically relevant concentrations (1 μM) on I_Na_ and I_K1_ recorded in commercial (iCell2^®^) human cardiomyocytes derived from induced pluripotent stem cells (hiPSC-CMs) and heterologous transfection systems. Our results demonstrated that these SGLT2is markedly increase both I_Na_ and I_K1_ densities in healthy hiPSC-CMs. The ultimate mechanisms underlying this unexpected effect and its possible therapeutic impact merit further analysis.

## 2. Materials and Methods

### 2.1. hiPSC-CM Culture and Infection

iCell Cardiomyocytes2^®^ (iCell2^®^) (Cellular Dynamics, Madison, WI, USA) hiPSC-CMs were thawed and cultured in 35 mm dishes containing glass coverslips coated with 0.1% gelatin, following methods previously described [19,20]. One week after thawing, cells were incubated with drugs and subsequently used for current recordings. iCell2 are highly purified human cardiomyocytes derived from hiPSC cells through optimized differentiation and purification protocols. In the lots used in our study, more than 99% of the cells were cardiomyocytes. Although iCell2 are a mixture of spontaneously electrically active atrial-like, nodal-like, and ventricular-like myocytes, the differentiation procedure resulted in a relatively homogeneous population of ventricular-like cardiomyocytes [19,20]. 

### 2.2. Chinese Hamster Ovary (CHO) Cell Culture and Transfection

Human Kir2.1 (NM_000891.2) (kindly provided by Dr. José Antonio SánchezChapula, Colima University, Colima, Mexico) was subcloned into a pcDNA3.1 plasmid (Invitrogen). Human cardiac Nav1.5 (hH1; NM_198056.2) and Navβ1 (NM_001037.4) cDNA, subcloned in a pCGI vector (which included GFP), was kindly gifted by Dr. Connie R. Bezzina (University of Amsterdam, The Netherlands). CHO cells were purchased from ATCC, and mycoplasma contamination was discarded through specific testing. CHO cells were cultured as previously described [20] and transiently transfected with cDNA-encoding Nav1.5 channels (1.6 µg) and Navβ1 (1.6 µg) (Nav1.5-β) or with Kir2.1 (1.6 µg) plus the cDNA encoding the CD8 antigen (0.5 µg); this was carried out through use of FUGENE XtremeGENE (Roche Diagnostics, Switzerland), with manufacturer instructions followed. At the point of 48 h after transfection, cells were incubated with polystyrene microbeads precoated with anti-CD8 antibodies (Dynabeads M450; Life Technologies, Carlsbad, CA, USA). Most of the cells that were beaded also exhibited channel expression. 

### 2.3. Ca^2+^/Calmodulin-Dependent Kinase II (CaMKII) Silencing in CHO Cells

For CaMKII silencing, CHO cells were transfected with four different short-inhibitory RNA (siRNA) duplexes (100 nM) or with scrambled siRNA (Sigma, San Luis, MO, USA) with use of Lipofectamine 2000 (Invitrogen, Waltham, MA, USA), according to manufacturer instructions. The sequence of the siRNA duplexes against the main four CaMKII isoforms expressed in CHO cells was the following: CaMKIIα [NM_177407.4] sense: 5′-GUUCCAGCGUUCAGUUAAU-3′; antisense: 5′-AUUAACUGAACGCUGGAAC-3′. CaMKIIβ [NM_001174053.1] sense: 5′-CUCAUUUGAGCCUGAAGCU-3′; antisense: 5′-AGCUUCAGGCUCAAAUGAG-3′. CaMKIIδ [NM_001025438.1] sense: 5′-CGUAAAGAUCCUUAUGGAA-3′; antisense: 5′-UUCCAUAAGGAUCUUUACG-3′. CaMKIIγ [NM_178597.5] sense: 5′-CUGUAACACCACUACAGAA-3′; antisense: 5′-UUCUGUAGUGGUGUUACAG-3′. Silencing with these constructions was previously demonstrated through Western blot by our group [19]. Current recordings were performed 24 h after siRNA transfection.

### 2.4. Patch-Clamp Recordings

Currents were recorded at room temperature (21–23 °C) by means of the whole-cell patch-clamp technique using Axopatch-200B patch-clamp amplifiers (Molecular Devices, San Jose, CA, USA) [19,20]. Recording pipettes were pulled from 1.0 mm o.d. borosilicate capillary tubes (GD1, Narishige Co., Ltd., Tokyo, Japan) via a programmable patch micropipette puller (Model P-2000 Brown-Flaming, Sutter Instruments Co., Novato, CA, USA) and heat-polished with a microforge (Model MF-83, Narishige). Micropipette resistance was kept below 1.5 MΩ for I_Na_ or 2–4 MΩ for I_K1_ when filled with the internal solution and immersed in the external solution. In all experiments, series resistance was compensated manually using the series resistance compensation unit of the Axopatch amplifier; ≥80% compensation was achieved. The remaining access resistance values after compensation and cell capacitance were, respectively, 1.6 ± 0.7 MΩ and 66.1 ± 18 pF (n = 25) in hiPSC-CMs and 1.2 ± 0.9 MΩ and 12.1 ± 1.1 pF (n = 34) in CHO cells. Therefore, under our experimental conditions, no significant voltage errors (<5 mV) due to series resistance were expected with the micropipettes used. Capacitance was not modified (*p* > 0.05) by dapa (73.5 ± 22 pF, n = 18 in hiPSC-CMs and 12.7 ± 1.2, n = 28 in CHO cells) or empa (73.5 ± 26 pF, n = 16 in hiPSC-CMs and 10.5 ± 0.9 pF, n = 27 in CHO cells). To minimize contribution of time-dependent shifts of channel availability during I_Na_/I_Nav1.5_ recordings, all data were collected 5-10 min after establishment of the whole-cell configuration. Under these conditions, current amplitudes and voltage dependence of activation and inactivation were stable during the time of recording [19,20]. I_Na_/I_Nav1.5_ and I_K1_/I_Kir2.1_ recordings were sampled at 50 and 4 kHz, respectively; filtered at half the sampling frequency; and stored on the hard disk of a computer for subsequent analysis. Data were analyzed using pCLAMP software (Molecular Devices).

In each experiment, current amplitudes were normalized to membrane capacitance in order to obtain current densities. To minimize influence of expression variability, currents were recorded in a large number of cells obtained from at least 3 different hiPSC-CM or CHO cell batches. 

#### 2.4.1. Solutions

-hiPSC-CMs. The external solution for I_Na_ recordings contained 20 mM NaCl, 1.5 mM MgCl_2_, 1 mM CaCl_2_, 115 mM CsCl, 5 mM HEPES, 10 mM glucose, and 1 μM nifedipine (pH = 7.35 with CsOH). Recording pipettes were filled with an internal solution that contained 10 mM NaF, 110 mM CsF, 10 mM EGTA, 20 mM CsCl, and 10 mM HEPES 10 (pH = 7.35 with CsOH). The external solution used to record I_K1_ contained 148 mM NaCl, 1 mM MgCl_2_, 1.8 mM CaCl_2_, 5.4 mM KCl, 0.4 mM NaH_2_PO_4_, 15 mM HEPES, 11 mM glucose, and 5 μM nifedipine (pH = 7.35 with NaOH). Recording pipettes were filled with an internal solution that contained 148 mM KCl, 1 mM MgCl_2_, 5 mM EGTA, 2 mM Creatine, 5 mM Mg-ATP, 5 mM Phosphocreatine, and 5 mM HEPES (pH = 7.2 with KOH). 

-CHO cells. To record I_Nav1.5_ and I_Kir2.1_, CHO cells were perfused with an external solution that contained 136 mM NaCl, 4 mM KCl, 1.8 mM CaCl_2_, 1 mM MgCl_2_, 10 mM HEPES, and 10 mM glucose (pH 7.4 with NaOH). Recording pipettes were filled with an internal solution that contained 80 mM K-aspartate, 42 mM KCl, 10 mM KH_2_PO_4_, 5 mM MgATP, 3 mM phosphocreatine, 5 mM HEPES, and 5 mM EGTA (pH 7.2 with KOH) to record I_Kir2.1_ (Liquid junction potential (LJP) = −13.2 mV); or 10 mM NaF, 110 mM CsF, 20 mM CsCl, 10 mM HEPES, and 10 mM EGTA (pH 7.35 with CsOH) to record I_Nav1.5_. I_Kir2.1_ current–voltage (I–V) curves were corrected according to the calculated LJP between the pipette and the external solution [20,21,22].

hiPSC-CMs and transfected CHO cells were incubated for 24 h with empa (1 µM) or dapa (1 µM). In some groups of experiments, hiPSC-CMs were incubated with NHE-1 inhibitor cariporide (10 µM). After incubation, current recordings were performed using the external solutions described above, supplemented with each corresponding drug. Empa, dapa, and cariporide were dissolved in DMSO to yield 10 mM stock solutions. Further dilutions were carried out in culture media to obtain the desired final concentration. Every control solution contained the same solvent concentration as the test solution.

#### 2.4.2. Pulse Protocols and Analysis

To construct current–voltage relationships for I_Na_ and I_Nav1.5_ recorded in hiPSC-CMs and CHO cells, respectively, we applied 50 ms pulses in 5 mV increments from −120 mV to potentials between −90 and +40 mV (I_Na_) or between −80 and +50 mV (I_Nav1.5_). To construct inactivation–availability curves, I_Na_ and I_Nav1.5_ were recorded after application of 500 ms pulses from −120 mV to potentials between −140 and −20 mV in 10 mV increments, followed by a test pulse to −30 mV (I_Na_) or −20 mV (I_Nav1.5_). 

Conductance–voltage curves for I_Na_ and I_Nav1.5_ recorded in hiPSC-CMs and CHO cells were constructed through plotting of normalized conductance as a function of membrane potential. Conductance was estimated for each experiment with this equation:

G = I/(V_m_ − E_rev_)

where G is conductance at test potential V_m_, I represents the peak maximum current at V_m_, and E_rev_ is reversal potential. To determine E_rev_, I_Na_ density–voltage relationships obtained in each experiment were fitted to a function of this form:

I = (V_m_ − E_rev_)×G_max_ ×(1 + exp[V_m_ − V_h_]/k)^−1^

where I is the peak current elicited at test potential V_m_, G_max_ is maximum conductance, and k is the slope factor.

Inactivation curves for I_Na_ and I_Nav1.5_ were constructed through plotting of current amplitude recorded with the test pulse as a function of the membrane potential of the preceding pulse. A Boltzmann function was fitted to activation/conductance–voltage and inactivation curves to obtain the midpoint (V_h_) and slope (k) values of the curves. The window currents for I_Na_ recorded in hiPSC-CMs were represented as the enlarged portion of the overlapping area between activation and inactivation curves. We also calculated the probability of being within the window using the following equation: (1/{1 + exp[(V_hact_ − V)/k_act_]} × ((1 − C)/{1 + exp[(V − V_hinact_)/k_inact_]} + C), where V_hact_, V_hinact_, k_act_, and k_inact_ are mean midpoint and slope values of activation and inactivation curves, respectively, and C is the bottom value of the fit of inactivation curve with the Boltzmann equation [19]. In order to describe the time course of I_Na_ and I_Nav1.5_ decay, a biexponential analysis was used as an operational approach, fitting peak current traces with an equation of this form:

y = C + A_f_ × exp(−t/τ_f_) + A_s_ × exp(−t/τ_s_)

where τ_f_ and τ_s_ are fast and slow time constants, whereas A_f_ and A_s_ are amplitudes of each component of the exponential and C is the baseline value. To quantify the time course of current activation, a monoexponential function was fitted to the activation phase of peak current traces, yielding the time constant (τ_act_) that defined the process.

The protocol for recording I_K1_ consisted of 250 ms pulses from −40 mV to potentials ranging between −120 and −40 mV in 10 mV steps. For I_Kir2.1_, the protocol for obtaining I-V curves consisted of 250 ms pulses in 10 mV increments, from –60 mV to potentials between −120 and +20 mV. I_K1_ and I_Kir2.1_ current amplitudes were measured at the end of the pulse.

### 2.5. Statistical Analysis

Results are expressed as mean ± SEM. Statistical analysis was performed using GraphPad Prism 8. To compare data from ≥3 experimental groups, one-way ANOVA was used, followed by Tukey’s test. An unpaired two-sided t-test was chosen for comparison of data from 2 experimental groups. In small-size samples (n < 5), statistical significance was confirmed using nonparametric tests (two-sided Wilcoxon’s test). To take repeated sample assessments into account, data were analyzed with multilevel mixed-effects models. Normality assumption was verified using the Shapiro–Wilk test. Variance was comparable between groups throughout the manuscript. We chose appropriate tests according to data distributions. A value of *p* < 0.05 was considered significant. For different groups of experiments, sample size was chosen empirically, according to previous experience in calculation of experimental variability. No statistical method was used to predetermine sample size. No particular procedure was followed for randomization/allocation of respective experimental groups.

## 3. Results

**Figure 1:**
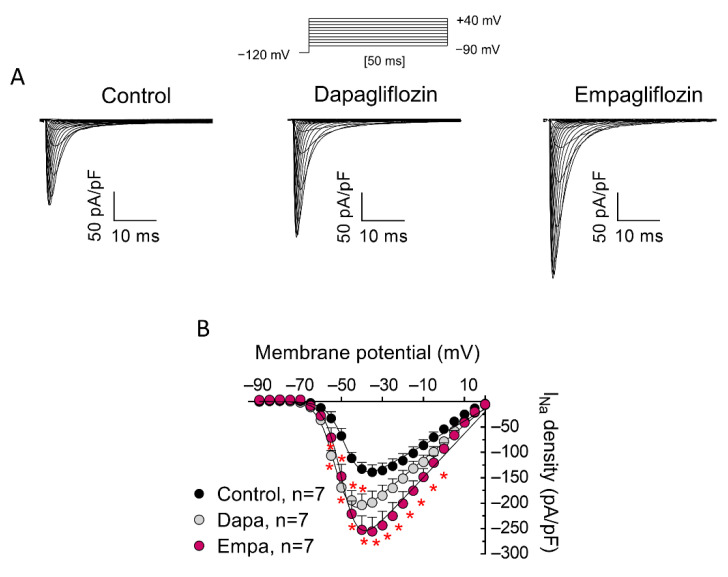
Incubation with dapa and empa increased I_Na_ recorded in hiPSC-CMs. (**A**) I_Na_ traces recorded, using the protocol shown at the top, in three different hiPSC-CMs: incubated with dapa or empa (1 µM, 24-h) or not incubated (control). (**B**) Current density-voltage relationships for peak I_Na_ recorded in hiPSC-CMs incubated with dapa or empa or not incubated. In (**B**), each point is the mean ± SEM of the “n” experiments indicated in the figure. * *p* < 0.05 vs. control.

As mentioned, a previous report demonstrated that acute application of 10 μM empa does not modify peak I_Na_ at any stimulation frequency [10]. Moreover, acute application [10] or incubation (for 24 h or 30 min) [8,9] with 1 μM empa significantly decreased I_NaL_. Therefore, we decided to test effects produced by incubation with 1 μM dapa and empa in order to allow a comparison of our data with those reported previously for this concentration. Figure 1A shows I_Na_ traces recorded in three different hiPSC-CMs, incubated with dapa or empa for 24 h or not incubated (control), while Figure 1B shows current density (peak current amplitude normalized to cell capacitance)-voltage relationships for the three groups of experiments. Incubation with both drugs significantly increased peak I_Na_ density in a wide range of membrane potentials. Maximum peak I_Na_ in control conditions and after incubation with dapa or empa averaged −139 ± 14, −203 ± 22 and −255 ± 28 pA/pF, respectively (n = 7, *p* < 0.05). Neither dapa nor empa altered reversal potential (E_rev_) of the current, time course of activation, or that of current decay (Table 1).

**Figure 2:**
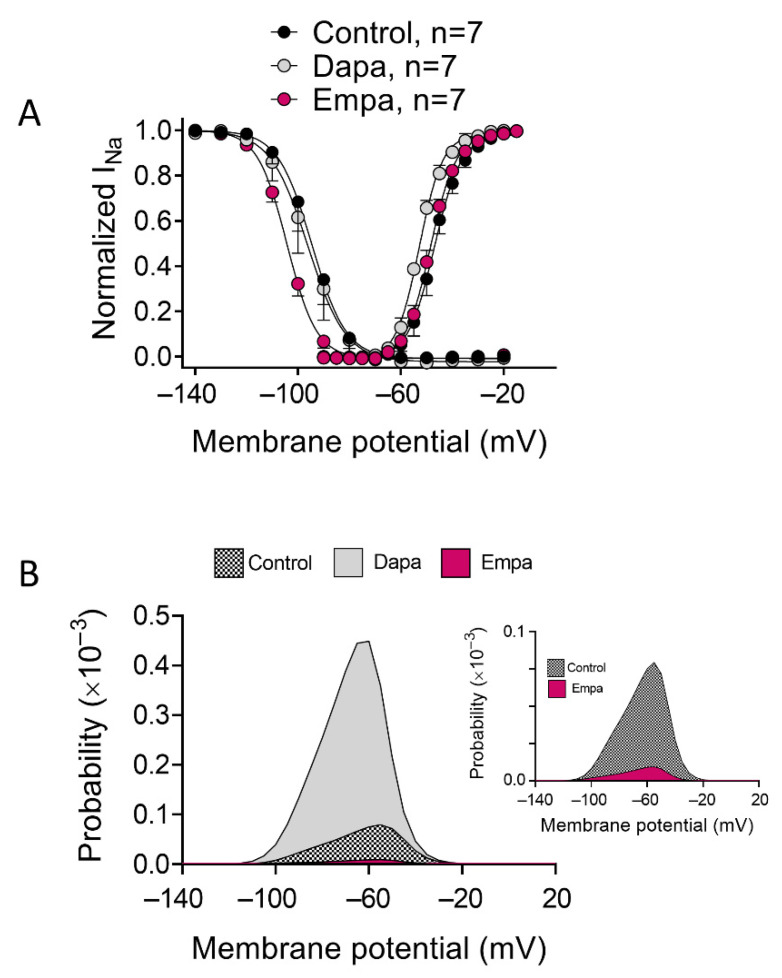
Dapa and empa modified voltage dependence of I_Na_ activation and inactivation, as well as window current. (**A**) Activation and inactivation curves for I_Na_ recorded in the three experimental groups. The solid lines represent the fit of a Boltzmann function. Each point is the mean ± SEM of the “n” experiments indicated in the figure. (**B**) Probability of being within the window current of hiPSC-CMs incubated with dapa or empa or not incubated. Probability of being within that window current for control and empa is also shown in the inset on an enlarged scale.

To determine the actions of SGLT2is on voltage dependence of activation, conductance-voltage relationships were constructed from density-voltage data as described in Methods where V_hact_ and k_act_ are the mean midpoint and slope values of the activation curves. Dapa, but not empa, shifted V_hact_ to more negative potentials (n = 7, *p* < 0.05) (Table 1 and Figure 2A) without modifying the k_act_. To identify possible drug effects on voltage dependence of channel availability, steady-state inactivation curves were constructed using a standard double-pulse protocol, as described in Methods. As can be observed in Figure 2A, empa, but not dapa, shifted V_h_ of inactivation curves to more negative potentials (n = 7, *p* < 0.05) without modifying the slope (*p* > 0.05) (Table 1). We next determined the effects of dapa and empa on the window current. The overlap of activation and steady-state inactivation of Na^+^ channels identified a range of voltages (i.e., window) where those channels had a small probability of being partially but not fully inactivated (Figure 2A). We also calculated the probability of being within the window defined by the product of the activation and steady-state inactivation parameters (Figure 2B). As a consequence of their effects on voltage dependence of activation and inactivation, dapa and empa markedly increased and decreased, respectively, window current and probability of being within the window (Figure 2B). 

**Figure 3:**
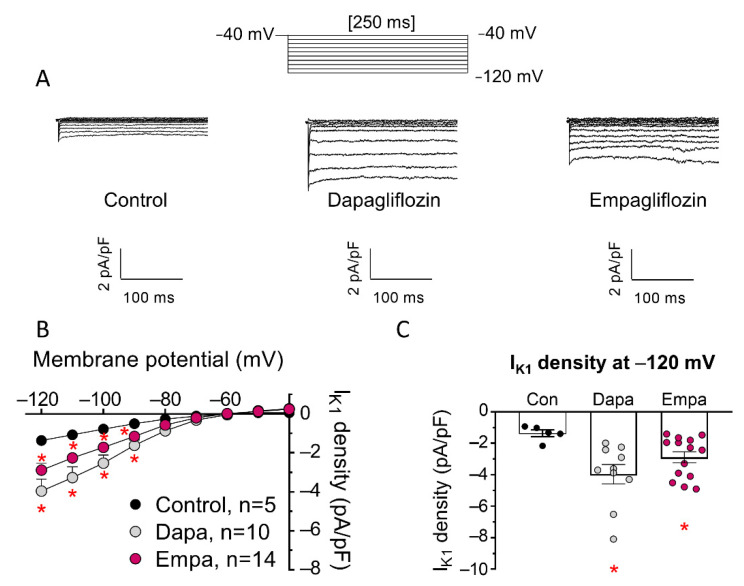
Incubation with dapa and empa increased I_K1_ recorded in hiPSC-CMs. (**A**) I_K1_ traces recorded in three different hiPSC-CMs: incubated with dapa or empa (1 µM, 24-h) or not incubated (control). (**B**,**C**) Current density-voltage relationships (**B**) and mean density for I_K1_ recorded at −120 mV (**C**) in hiPSC-CMs incubated with dapa or empa or not incubated. In B, each point is the mean ± SEM of the “n” experiments indicated in the figure, and in (**C**), each bar shows the mean ± SEM of the “n” experiments, and each point represents one experiment. * *p* < 0.05 vs. control.

Figure 3A shows I_K1_ traces recorded in three different hiPSC-CMs, incubated with dapa or empa or not incubated, and Figure 3B,C show current density-voltage relationships for I_K1_ recorded in the three groups of experiments, as well as comparison of the mean density of the current recorded at −120 mV. Incubation with both drugs markedly increased cardiac I_K1_ density at potentials negative to E_rev_, with a ≈ two- to three-fold increase at −120 mV (Figure 3C).

**Figure 4:**
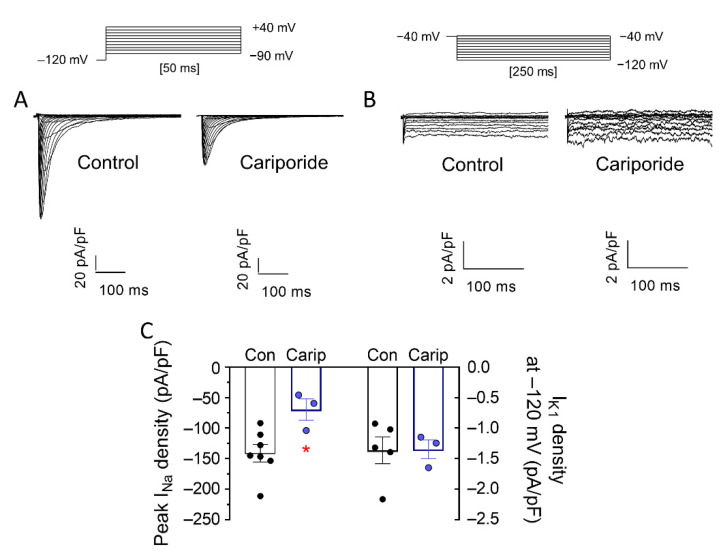
Inhibition of NHE-1 did not mediate increasing effects of dapa and empa on I_Na_ and I_K1_. (**A**,**B**) I_Na_ (**A**) and I_K1_ (**B**) traces recorded, using the protocol shown at the top, in four different hiPSC-CMs incubated with NHE-1 inhibitor cariporide (10 µM, 24-h) or not incubated (control). (**C**) Maximum densities of peak I_Na_ (left) or I_K1_ recorded at −120 mV (right) in cells incubated with cariporide or not incubated. In (**C**), each bar shows the mean ± SEM of the “n” experiments, and each point represents one experiment. * *p* < 0.05 vs. control.

It has been proposed that SGLT2is inhibit sodium–hydrogen exchanger-1 (NHE1) in cardiomyocytes and that this inhibition may underlie some cardiac effects attributed to these drugs [23], although this is still a matter of debate [24]. Therefore, if NHE1 inhibition were involved in the I_Na_ and I_K1_ increasing effects produced by dapa and empa, one would expect that a NHE1 inhibitor would produce identical effects. As can be observed in Figure 4, incubation of hiPSC-CMs with cariporide (24-h, 10 μM), a selective NHE1 inhibitor, significantly decreased I_Na_ but did not modify I_K1_ (Figure 4A,B). Therefore, these results strongly suggest that inhibition of NHE1 does not mediate increasing effects of dapa and empa on I_Na_ and I_K1_.

**Figure 5:**
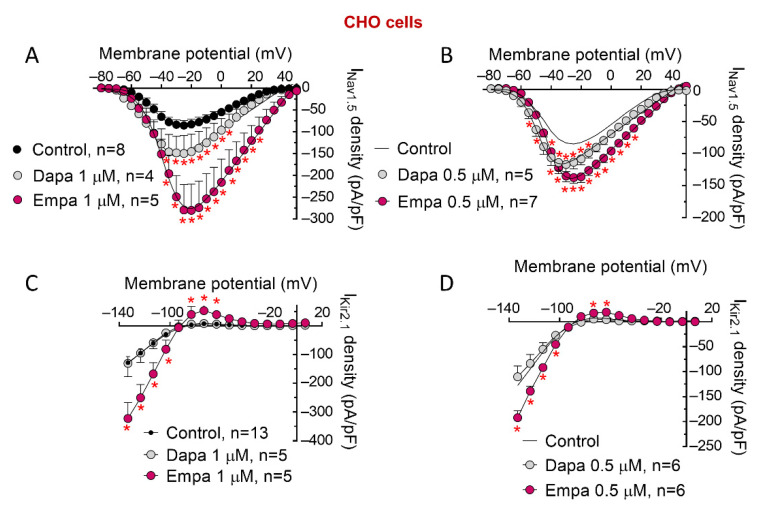
Effects of dapa and empa on I_Nav1.5_ and I_Kir2.1_ recorded in CHO cells. (**A**,**B**) I_Nav1.5_ density–voltage relationships for currents recorded in CHO cells transiently transfected with human Nav1.5 channels incubated with dapa or empa at 1 (**A**) or 0.5 µM (**B**) or not incubated. (**C**,**D**) I_Kir2.1_ density–voltage relationships for currents recorded in CHO cells transiently transfected with human Kir2.1 channels incubated with dapa or empa at 1 (**C**) or 0.5 µM (**D**) or not incubated. Each point is the mean ± SEM of the “n” experiments indicated in the figure. * *p* < 0.05 vs. control.

To get a deeper insight into the mechanism underlying increasing effects of these drugs, we explored their actions in CHO cells transiently transfected with human Nav1.5 or Kir2.1. It has been described that expression of Nav1.5 and Kir2.1 channels is reciprocally and positively modulated in such a way that augmented expression of Nav1.5 channels is accompanied by increased expression of Kir2.1 channels, and vice versa [12,20,22,25]. Therefore, we first tested whether I_Na_- and I_K1_-increasing effects produced by dapa and empa were secondary to this positive reciprocal modulation. To this end, Nav1.5- or Kir2.1-transfected CHO cells were incubated (24-h, 1 μM) with dapa or empa. The effects produced by empa and dapa on I_Nav1.5_ were nearly identical to those produced on I_Na_ in hiPSC-CMs; i.e., both increased I_Na_ density at different membrane potentials without modifying time-dependent parameters of peak currents (Figure 5A and Table 1). Furthermore, dapa and empa shifted to more hyperpolarized potentials the voltage dependence of Nav1.5 channel activation and inactivation, respectively (Table 1). We also tested effects produced by incubation of both SGLT2is at a lower concentration (0.5 μM). Figure 5B demonstrates that dapa and empa at this concentration significantly increased peak I_Nav1.5_ densities (n ≥ 5, *p* < 0.05).

With regards to I_Kir2.1_, empa, at the two concentrations tested, also significantly and markedly increased inward and outward components of the current (n ≥ 5, *p* < 0.05) (Figure 5C,D). Conversely, incubation with dapa, even at 1 μM, did not modify I_Kir2.1_ at any membrane potential (Figure 5C,D). 

**Figure 6:**
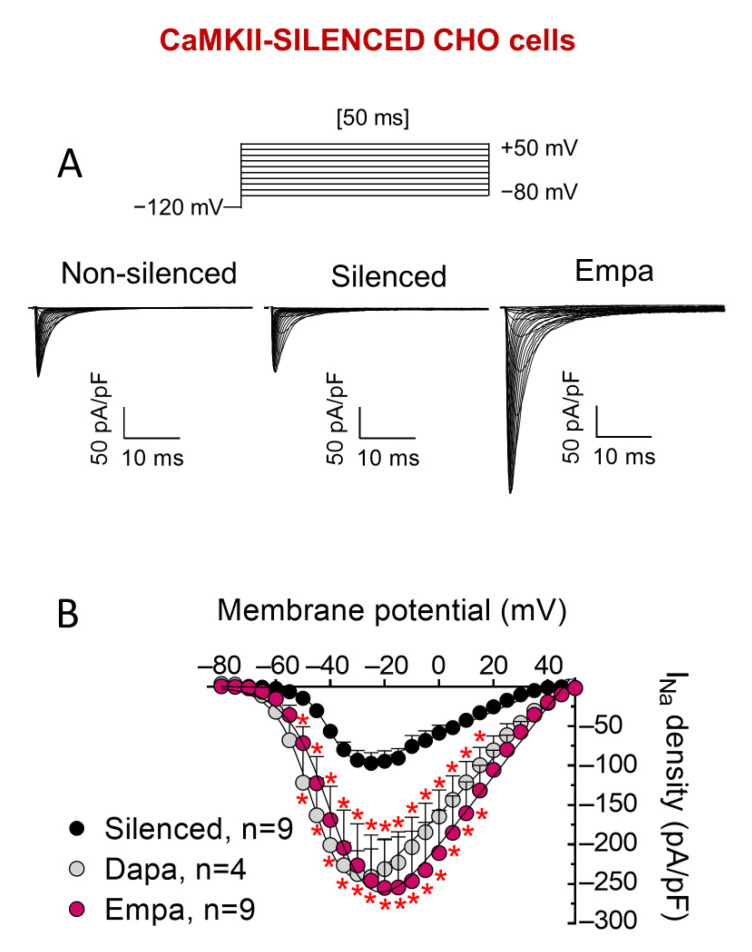
Neither dapa- nor empa-induced increase of I_Na_ was mediated by CaMKII. (**A**) I_Nav1.5_ traces recorded, using the protocol shown at the top, in a non-silenced CHO cell and in two different CHO cells in which CaMKII expression was reduced by 65% by means of specific siRNA. The latter cells were incubated with empa (1 µM, 24-h) or not (Silenced). (**B**) I_Nav1.5_ density-voltage relationships obtained in CAMKII-silenced CHO cells incubated with dapa or empa or not incubated. In (**B**), each point is the mean ± SEM of the “n” experiments indicated in the figure. * *p* < 0.05 vs. control.

It is well established that Nav1.5 channels are important targets of CaMKII in cardiac myocytes [26]. It has been shown that CaMKII phosphorylation of Nav1.5 leads to an increase of late I_Na_ (I_NaL_) and to a shift of voltage dependence of Nav1.5 channel inactivation to hyperpolarized potentials [27]. Interestingly, it has also been shown that empa and dapa inhibit CaMKII activity [28,29]. Therefore, to test whether CaMKII modulation by SGLT2is is responsible for I_Na_-increasing effects, we silenced CaMKII expression in CHO cells by means of specific siRNA [20]. As expected, CaMKII silencing resulted in a statistically significant acceleration of time course of inactivation, as well as a depolarizing shift of inactivation curves (Table 1). Moreover, as can be observed in Figure 6A,B and Table 1, I_Na_ increase and effects on voltage dependence of activation and inactivation, produced by 24 h incubation with both SGLT2is (1 μM), were also evident in CaMKII-silenced cells.

## 4. Discussion

Our results demonstrated that incubation with dapa and empa significantly increased densities of I_Na_ and I_K1_ in human cardiomyocytes derived from iPSC. Furthermore, these actions were independent of inhibitory activity of NHE1 and CaMKII, presumably produced by SGLT2is. Moreover, empa- and dapa-induced augmentation of I_Na_ and I_K1_ were not identical, suggesting that this is not a “class-effect” common to all SGLT2is.

As demonstrated by our experiments, dapa and empa significantly increase I_Na_ density in hiPSC-CMs and in CHO cells that transiently express human Nav1.5 + Navβ1 channels. Interestingly, both dapa and empa affected voltage dependence of I_Na_, albeit differentially, suggesting that there is no common mechanism underlying the effects. Dapa and empa shifted to more negative potentials the voltage dependence of I_Na_ activation and inactivation, respectively. The hyperpolarizing shift of the activation curve produced by dapa would contribute to I_Na_ augmentation. Conversely, the negative shift of the inactivation curve produced by empa would diminish Na^+^ channel availability as well as I_Na_. As a consequence of these effects, dapa augmented the probability of being in the window current while empa diminished it. Importantly, an increase of the window current would enhance I_NaL_ [30]. Accordingly, a window-current decrease produced by empa would decrease I_NaL_ [30]. 

A single increase in Nav1.5 subunits at the sarcolemma would enhance current density without any modification in the current gating. For instance, recent results demonstrated that pro-transcriptional effects produced by the Tbx5 transcription factor over the promoter of the *SCN5A* gene (which encodes Nav1.5 channels) increase I_Na_ without modifying its voltage dependence. This is carried out through augmentation of presence of Nav1.5 channels at the membranes of cardiomyocytes [19]. Therefore, it seems reasonable to assume that I_Na_ increase produced by both SGLTis is not a consequence of an augmentation of the number of Nav1.5 subunits at the sarcolemma, or at least not exclusively. 

Both dapa and empa increase I_K1_ density in hiPSC-CMs at negative membrane potentials. Unfortunately, the outward component of I_K1_ is hardly observed in hiPSC-CMs because these cells are largely immature. As mentioned, it is well-documented that Nav1.5 and Kir2.1 channel expression is reciprocally and positively modulated [12,20,22,25]. This modulation is complex, since multiple processes of Nav1.5 and Kir2.1 channel biology, including forward trafficking from early stages after channel synthesis, internalization, and degradation, are affected [12,20,22,25,31]. To dissect whether the simultaneous augmentation of I_Na_ and I_K1_ observed in hiPSC-CMs was due to reciprocal modulation, we conducted experiments in CHO cells that separately expressed Nav1.5 or Kir2.1 channels. In CHO cells expressing Kir2.1 channels alone, incubation with empa markedly and significantly increased I_K1_, while dapa incubation did not. These results suggest that dapa-induced I_K1_ increase in hiPSC-CMs is secondary to augmentation of expression of Nav1.5 channels. Conversely, an empa-induced I_K1_ increase resulted from a direct empa effect on Kir2.1 channels, since it was also apparent in CHO cells that did not express Nav1.5 channels. Furthermore, empa significantly increased I_K1_ both at very negative and at membrane potentials between −80 and −60 mV, i.e., at a physiological range of membrane potentials. Further experiments will be needed to test whether these SGLT2is hyperpolarized the resting membrane potential. Should this occur, empa and dapa would increase excitability of cardiac myocytes.

Previous data support that empa incubation suppresses CaMKII activity in isolated ventricular cardiomyocytes [28]. As mentioned, CaMKII is an important I_Na_ regulator, since CaMKII-induced phosphorylation of Nav1.5 channels increases I_NaL_, slows fast I_Na_ inactivation and reactivation, and shifts voltage-dependent inactivation to more negative potentials [26]. Empa decreased I_NaL_ in mouse cardiomyocytes obtained from HF models with reduced [10] and preserved [9] ejection fraction, as well as in ventricular myocytes from patients with aortic stenosis [8]: effects that were attributed to an empa-induced CaMKII inhibition [8,9]. Our results suggest that the hyperpolarizing shift of the inactivation curve produced by empa could contribute, at least partially, to I_NaL_ reduction via decrease of the window current. Moreover, we demonstrated that both dapa and empa actually increase I_Na_ density in CaMKII-silenced CHO cells: a result that strongly suggests that the effects described herein were not related to CaMKII modulation produced by SGLT2is. 

Several reports support the contention that acute exposure of empa inhibits NHE1 activity in human and rodent cardiomyocytes [23,32,33] and that this effect is responsible for a decrease of intracellular Na^+^ concentration ([Na^+^]_i_). Other authors propose that empa modifies neither NHE1 activity nor [Na^+^]_i_ [24]. Independently of this controversy, we tested whether NHE1 inhibition produces similar effects on I_Na_ to those produced by dapa and empa. Our results showed that incubation with NHE1 inhibitor cariporide did not increase but decreased I_Na,_ nor did it modify I_K1_ density. Therefore, possible inhibition of NHE-1 produced by SGLT2is does not mediate increasing effects, produced by empa and dapa, on I_Na_ and I_K1_.

Overall, our results suggest that dapa and empa directly increase I_Na_ and that empa also directly increases I_K1_. Current density depends on the number of channels present at the sarcolemma and the channel conductance. On the other hand, the number of channels depends on the balance between trafficking pathways to and from the cell membrane. Ongoing experiments in our laboratory will discriminate whether dapa and empa modify steady-state Nav1.5 channel density and/or conductance: two mechanisms that are not mutually exclusive. Several drugs increase expression of ion channels at the sarcolemma, thus acting as chaperone-like molecules (pharmacoperones). For instance, flecainide increases expression of Kir2.1 channels [21], while several hERG channel blockers (E-4031), as well as lumacaftor and ivacaftor, promote expression of hERG subunits in the cell membrane [34]. Moreover, amiodarone and its analogue dronedarone increase functional expression of Kir2.1 channels by impairing Kir2.1 backward trafficking [35]. Therefore, it could be possible that dapa and empa exert a chaperone-like effect by increasing expression and forward trafficking of pore-forming subunits and/or by decreasing backward trafficking (internalization and degradation) of channel subunits. Nowadays, there is an increasing interest in possible therapeutic utility of pharmacoperones for treatment of inherited cardiac arrhythmia, since ion channel mutations often lead to disorders in trafficking of health and mutated proteins [36]. Defects in channels’ protein trafficking are responsible for electrical remodeling that enhances risk of acquired arrhythmia, such as those associated with HF. Therefore, it seems reasonable to propose that pharmacoperones could also be useful for treatment of acquired arrhythmias.

One limitation of our experiments was that for hiPSC-CMs, we tested a single dose of dapa and empa, each of which was incubated only for one time point. We selected this concentration of dapa and empa to allow a comparison of our data with those reported previously for empa. It has to be taken into consideration that we added dapa and empa to the cell culture medium that contained serum, allowing the drugs to bind to plasma proteins. Therefore, in our experiments, expected free dapa concentrations were around 0.05 and 0.1 μM (50 and 100 nM), considering a 90% binding to plasma proteins [37,38]. After administration of a single dose of 5 or 25 mg of dapa, Cmax reached ranged 66–70 and 279–283 ng/mL, respectively [37,38]. Free (unbounded) peak plasma concentration would be around 16.5 and 68.5 nM, respectively. Importantly, the area under the curve (AUC) for 5 and 25 mg doses was 220–228 and 1037–1072 ng·h/mL, respectively [37,38]. On the other hand, after a single dose of 10 or 25 mg of empa, Cmax ranged 211–226 and 505–692 nM [39,40], which, considering an 86% binding to plasma proteins, would yield free peak plasma concentrations of around 30 and 71–97 nM, respectively. In this case, AUC for 10 and 25 mg doses were 1730 and 3830–4300 ng·h/mL, respectively [39,40]. Importantly, Cmax and AUC increased after administration of multiple doses of both SGLT2is. Overall, these data strongly support the contention that the dapa and empa concentrations tested here (0.5 and 1 μM) can be considered therapeutically relevant.

## 5. Conclusions

In summary, we demonstrated that incubation with dapa and empa significantly increases I_Na_ and I_K1_ in human healthy cardiomyocytes through an unknown mechanism that is currently under analysis. We propose that empa and dapa could represent a new class of drugs with a novel and long-pursued antiarrhythmic mechanism of action. The key will be to test whether these actions are also apparent in myocardia of HF patients and whether they may improve HF- induced electrical remodeling. Should this occur, our hypothesis is that I_Na_- and I_K1_-increasing effects produced by empa and dapa would definitively contribute to cardioprotective effects.

## Figures and Tables

**Table 1:** Effects of dapa and empa on time and voltage dependence of peak I_Na_ activation and inactivation. CHO = Chinese hamster ovary. hiPSC-CMs = human-induced pluripotent stem cell-derived cardiomyocytes. τact = time constant of activation yielded from fit of a monoexponential function to peak maximum current. τ_finact_ and τ_sinact_ = fast and slow time constants of inactivation yielded from fit of a biexponential function to peak maximum current decay. V_hact_ and *k*_act_ = midpoint and slope values of conductance-voltage curves; V_hinact_ and *k*_inact_ = midpoint and slope values of inactivation curves. Each value represents mean ± SEM of >6 cells/experiments from at least three different dishes in each group. * *p* < 0.05 vs. control. # *p* < 0.05 vs non-silenced CHO cells. ANOVA followed by Tukey’s te.

hiPSC-CMs									
		Activation						Inactivation	
	τ_act_ (ms)	V_h_ (mV)	*k*	τ_finact_ (ms)	A_finact_ (%)	τ_sinact_ (ms)	A_sinact_ (%)	V_h_ (mV)	*k*
Control	0.27 ± 0.02	−47.2 ± 1.6	4.7 ± 0.4	1.5 ± 0.1	83.1 ± 3.7	6.1 ± 0.9	16.9 ± 3.7	−97.3 ± 4.5	5.6 ± 0.2
Dapa	0.29 ± 0.01	−55.5 ± 2.8 *	4.6 ± 0.6	1.7 ± 0.1	85.3 ± 2.9	8.2 ± 1.3	14.7 ± 2.9	−96.2 ± 4.3	5.7 ± 0.4
Empa	0.27 ± 0.03	−48.5 ± 0.8	4.5 ± 0.3	1.5 ± 0.1	83.2 ± 1.7	6.0 ± 0.5	16.8 ± 1.7	−108.6 ± 4.4 *	5.4 ± 0.3
CHO cells									
	Activation			Inactivation					
	τ_act_ (ms)	V_h_ (mV)	*k*	τ_finact_ (ms)	A_finact_ (%)	τ_sinact_ (ms)	A_sinact_ (%)	V_h_ (mV)	*k*
Control	0.22 ± 0.02	−37.6 ± 2.5	6.3 ± 0.4	1.3 ± 0.1	86.5 ± 1.3	8.8 ± 1.1	13.5 ± 1.3	−81.5 ± 2.2	5.7 ± 0.3
Dapa	0.22 ± 0.01	−45.7 ± 3.1 *	6.2 ± 0.4	1.5 ± 0.1	88.7 ± 1.1	9.2 ± 1.2	11.3 ± 1.1	−83.7 ± 4.4	5.8 ± 0.4
Empa	0.21 ± 0.01	−40.4 ± 4.8	6.3 ± 0.8	1.3 ± 0.05	85.5 ± 2.7	5.7 ± 0.7	14.5 ± 2.7	−94.5 ± 0.8 *	6.1 ± 0.4
CaMKII-silenced CHO cells									
	Activation			Inactivation					
	τ_act_ (ms)	V_h_ (mV)	*k*	τ_finact_ (ms)	A_finact_ (%)	τ_sinact_ (ms)	A_sinact_ (%)	V_h_ (mV)	*k*
Control	0.25 ± 0.03	−40.6 ± 1.8	6.3 ± 0.7	1.0 ± 0.08 #	82.7 ± 2.8	6.9 ± 0.6 #	17.3 ± 2.8	−76.2 ± 1.5 #	5.4 ± 0.6
Dapa	0.23 ± 0.03	−48.8 ± 3.5 *	6.1 ± 0.7	1.2 ± 0.1 #	83.7 ± 2.9	5.4 ± 0.8 #	16.3 ± 2.9	−76.9 ± 4.1 #	5.7 ± 0.1
Empa	0.22 ± 0.02	−38.6 ± 1.0	6.5 ± 0.5	1.1 ± 0.1 #	85.0 ± 2.4	6.5 ± 0.5	15.0 ± 2.4	−91.4 ± 2.2 *#	5.5 ± 0.3

## Data Availability

Data will be available upon reasonable request to the corresponding author.
